# Effect of Milk Replacer Feeding Level and Concentration on Feed Utilization, Growth Performance, and Blood Parameters of Hanwoo Calves

**DOI:** 10.3390/ani14233487

**Published:** 2024-12-03

**Authors:** Wangyong Jeong, Sang Rak Lee, Youngjun Na, Yongjun Choi

**Affiliations:** 1 Department of Animal Science and Technology, Konkuk University, Seoul 05029, Republic of Korea; jwangyong@gmail.com (W.J.); leesr@konkuk.ac.kr (S.R.L.); 2 Animal Data Laboratory, Antller Inc., Seoul 05029, Republic of Korea; ruminoreticulum@gmail.com; 3 School of Animal Life Convergence Science, Hankyong National University, Anseong 17579, Republic of Korea

**Keywords:** concentration, feeding level, growth performance, Hanwoo calves, milk replacer

## Abstract

This study aims to determine appropriate milk replacer feeding amounts and concentrations for artificial suckling of Hanwoo calves by analyzing growth and blood parameters in response to varying feeding levels and concentrations. A total of 90 Hanwoo calves (45 females; 45 males) (Mean weight 30.84 (3.61) kg; females 29.22 (3.47) kg; males 32.46 (2.99) kg) were allocated in the experiment. The treatment design consisted of a 3 × 3 factorial arrangement, resulting in 9 treatment groups based on milk replacer feeding levels and concentrations. Milk replacer feeding levels were set at 10, 20, and 30% of calf body weight, while concentrations were 15, 20, and 25%. The blood parameters, average daily gain, feed efficiency, diarrhea incidence, feed intake, and water intake were measured. Optimal feed intake was observed at milk replacer levels above 4.0% DM of BW (20% of body weight × 20% concentration) (*p* < 0.05). Maximum average daily gain was achieved with 4.5% DM of BW (30% of body weight × 15% concentration). The 25% milk replacer concentration showed reduced growth performance and increased diarrhea incidence compared with lower concentrations. Blood parameters remained unaffected across treatments. For 30-day-old Hanwoo calves, the ideal feeding regimen appears to be 4.5% DM of BW (30% of body weight × 15% concentration) divided into four daily feedings. However, for younger calves or when digestive issues are a concern, a two-phase approach is recommended: 3.0% DM of BW (20% of body weight × 15% concentration) for the first 10 days, followed by an increase to 4.5% DM of BW (30% of body weight × 15% concentration) thereafter. This feeding strategy aims to optimize growth performance while minimizing potential digestive disturbances during the critical early development stage of Hanwoo calves.

## Simple Summary

This study investigated the effects of milk replacer feeding levels and concentrations on Hanwoo calf performance. Optimal feed intake was observed at milk replacer levels above 4.0% DM of BW (20% of body weight × 20% concentration). Maximum average daily gain was achieved with 4.5% DM of BW (30% of body weight × 15% concentration). The 25% milk replacer concentration showed reduced growth performance and increased diarrhea incidence compared with lower concentrations. Blood parameters remained unaffected across treatments. This feeding strategy suggests guidelines to optimize growth performance while minimizing potential digestive disturbances during the critical early development stage of Hanwoo calves.

## 1. Introduction

As beef farms improve their breeding techniques and calf-rearing skills, producing higher-quality calves significantly impacts farm profitability. The dam’s nursing ability influences the calf’s growth, disease resistance, and immunity during both the nursing period and post-weaning [1]. The health and quality of calves greatly affect their milk production capacity as adults [2] and the carcass characteristics of beef cattle [3], emphasizing the importance of proper calf rearing. The average body weight of Hanwoo calves was reported to be 30.47 kg and 28.16 kg for males and females, respectively [4]. The goal of average daily gain is recommended to be 0.6 to 1.0 kg in the guideline of the Korean feeding standard for Hanwoo [5]. Hanwoo cows have low milk production, with approximately 3.53 kg/d during 4 months after farrowing, and when the weight of a Hanwoo calf exceeds 40 kg, the nutrients obtained from maternal milk alone become insufficient to achieve the target daily weight gain [5].

For Hanwoo calves, weaning is recommended at approximately 3 months of age, typically when the calf reaches a weight of around 80 kg [6]. Because nutrients are lacking during suckling periods of Hanwoo calf, artificial suckling techniques and research have long been performed for dairy cattle, with standardized pre- and post-weaning management practices [7]. However, Hanwoo calves are typically raised using natural suckling methods by the dam, with research focusing on maximizing calf growth during natural suckling and optimizing starter feed intake post-weaning [1,8,9]. As farming techniques become more developed, interest in the artificial suckling of Hanwoo calves using milk replacers is growing. However, due to the scarcity of artificial suckling experiments with Hanwoo calves, most references are based on dairy calf studies or Japanese calf research.

To maximize calf intake and growth during artificial suckling, it is crucial to determine appropriate milk replacer feeding levels. While increasing milk supply in early life can enhance growth rates, it may lead to reduced starter feed intake at weaning [10]. The milk replacer feeding method of 10% of body weight, which is widely used in calf suckling, results in lower growth rates compared with naturally suckled calves [11]. To improve the growth of calves, strategies to increase milk solids beneficial for calf growth include feeding them milk with higher solid concentration, adding extra solids to milk, or increasing milk volume [12]. With the recent convergence of information and communication technology (ICT) and livestock farming technologies, there is an increasing demand for automation to simplify animal husbandry methods. This integration of technologies into livestock management has the potential to enhance calf husbandry methods, improving efficiency. The development of precise feeding protocols for Hanwoo calves, particularly regarding milk replacers, can serve as a crucial stepping stone towards the implementation of automated feeding systems. Some studies report the probability of milk replacer in beef calf growth [13]; however, research on milk replacer concentration levels is limited, with most studies dating back to the 1970s or earlier. Furthermore, most studies primarily focus on dairy breeds, with no experimental research on Hanwoo calves.

Therefore, this study aims to determine appropriate milk replacer feeding amounts and concentrations for the artificial suckling of Hanwoo calves by analyzing growth and blood parameters in response to varying feeding levels and concentrations.

## 2. Materials and Methods

The research protocols of this study were performed according to the Konkuk University Animal Care and Use Committee (Approval number: KU21065).

### 2.1. Animal Care

This experiment was conducted at Seowoo farm in *Jeongeup*, *Jeollabuk-do*, from 25 April to 26 September 2021 (mean temperature 19.7 (6.3) °C, relative humidity 74.5 (9.7)%) for a total of 155 days. A total of 90 Hanwoo calves (0 d old; 45 females; 45 males) (mean weight 30.84 (3.61) kg; females 29.22 (3.47) kg; males 32.46 (2.99) kg) were allocated in the experiment after birth. After birth, the calves’ navels were disinfected, and within 12 h, they were fed commercial powdered colostrum (F1RST start 60, ASP La Belle, Phoenix, AZ, USA) mixed with warm water at 250 g/L and were fed an intake of over 100 g IgG per day over two feedings. The calves received 2 mL of a vitamin A, D3, and E supplement (Vigantol-E [Vitamin A, 300,000 IU; Vitamin D3, 100,000 IU; Vitamin E, 50 mg]; Elanco Co., Greenfield, IN, USA), 1 mL of a selenium preparation (Selevit, Fatro, Canada), and 1 mL of trimethoprim (Amphoprim, Virbac, Carros, France) for disease protection. And, within a week, they were also administered a 2 mL respiratory vaccine (Inforce 3, Zoetis, Parsippany-Troy Hills, NJ, USA) intranasally to prevent respiratory diseases caused by bovine infectious rhinotracheitis, parainfluenza virus 3, and respiratory syncytial virus according to the method of Ellis et al. [14].

**Figure 1:**
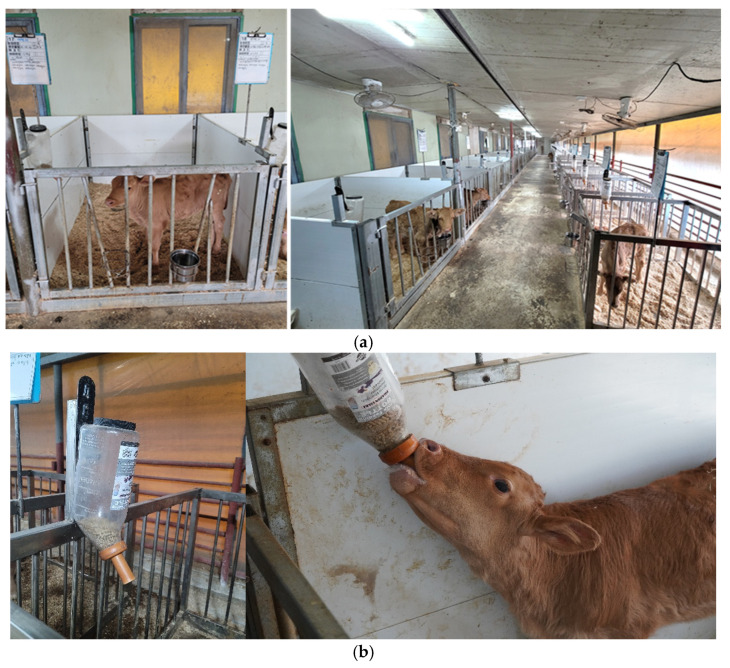
Experimental Hanwoo calf sawdust bedded pack barn: (**a**) sawdust bedded pack barn; (**b**) grain feeding bottle.

The experimental housing was a naturally ventilated, open-sided barn with winch curtains on one wall, protecting calves from direct exposure to the external environment (Figure 1a). Calves were housed in individual pens equipped with climate control to maintain indoor temperatures between 15–25 °C (Figure 1a; length × width × height, 1.33 m × 1.85 m × 1.00 m). Each pen door had frames for securing water and feed containers. A grain feeding bottle (Figure 1b; Grain feeding bottle, Coburn, South Windso, WI, USA) was installed on the right wall at a height of 80 cm for easy access to starter feed without external contamination. Feed in the grain bottle and water in the drinking container were replaced daily.

Following the farm’s standard rearing program, sawdust bedding was provided at a depth of 5–6 cm for calf comfort and warmth. Bedding was visually inspected daily at 9:00 A.M. and completely replaced if approximately 50% or more was contaminated with excreta. When necessary, only the contaminated areas were supplemented with fresh sawdust.

### 2.2. Experimental Design

Animals were allocated to have similar average body weights among experimental units. The treatment design consisted of a 3 × 3 factorial arrangement, resulting in 9 treatment groups, each replicated 10 times based on milk replacer feeding levels and concentrations (Table 1; Nukamel Yellow, NuKamel, Olen, Belgium).

Milk replacer feeding levels were set at 10, 20, and 30% of calf body weight, while concentrations were 15, 20, and 25%. Each calf served as an experimental unit, and results were analyzed individually (Table 2).

All calves were fed milk replacer four times daily using 2.5 L bottles equipped with artificial teats (diameter 2 cm; length 7.5 cm). Bottles were cleaned and dried after each feeding. Milk replacer was offered at 6:00, 11:00, 16:00, and 21:00 daily, with any remaining amounts recorded. The milk replacer powder was mixed with 42 °C warm water to achieve the appropriate concentration for each treatment group before feeding. Precise amounts were measured using a measuring cup and poured into the feeding bottles.

Calves were allowed to drink freely until they exhibited satiety behaviors or refused further intake. No forced feeding was implemented. Starter feed (Table 1; Chunhajeilfeed, Daejeon, Republic of Korea) was replaced daily at 16:00 after the afternoon milk feeding. From day 0 to day 30 of the experiment, starter feed was provided *ad libitum* in designated feed containers. Water was supplied in stainless steel buckets starting from the beginning of the experiment, coinciding with feed provision times, and was replaced with fresh water daily. Milk replacer was provided in restricted quantities with no residuals, and the amounts of water and remaining feed were measured before providing fresh feed the following day. Because the experiment was conducted using newborn calves, pen acclimation was not performed.

### 2.3. Chemical Composition

The dry matter (DM), ash, and ether extract (EE) content of feed and milk replacer were analyzed according to the methods of AOAC [15]. The DM content was determined by drying samples in a forced-air oven (HB-503-LF, Hanbaek Scientific Technology, Bucheon-si, Republic of Korea) at 60 °C for 48 h. Ash content was measured by incineration in an electric muffle furnace (Isotemp muffle furnace 550–126, Thermo Fisher Scientific Inc., Waltham, MA, USA) at 550 °C for 3.5 h. The organic matter (OM) content was calculated by subtracting the ash weight from the dry matter content. EE was analyzed using an ANKOM HCl Hydrolysis System (ANKOM Technology, New York, Macedon, NY, USA) following the method described by Mertens and collaborators [16]. Crude protein (CP) content was determined using the Kjeldahl method (AOAC 968.06) with an automatic Kjeldahl analyzer (Kjeltec 8400, FOSS, Hilleroed, Denmark). Calcium and phosphorus concentrations were measured using inductively coupled plasma optical emission spectrometry (ICP-OES iCAP PRO, Thermo Fisher Scientific, Waltham, MA, USA) after appropriate sample preparation.

### 2.4. Blood Profiles

Common blood cell (CBC) analysis was performed on whole blood samples using a blood analyzer (VetScan HM2, Abaxis, Union City, CA, USA) to measure red blood cells (RBC), hematocrit (HCT), hemoglobin (Hb), mean cell volume (MCV), mean corpuscular hemoglobin (MCH), mean corpuscular hemoglobin concentration (MCHC), red cell distribution width (RDW), reticulocytes (RETIC), white blood cells (WBC), neutrophils (NE), lymphocytes (LY), monocytes (MO), eosinophils (EO), basophils (BA), platelets (PLT), mean platelet volume (MPV), platelet distribution width (PDW), and procalcitonin (PCT).

Serum metabolite analysis was conducted using a clinical chemistry automated analyzer (FUJI DRI CHEM 7000i biochemistry analyzer, Fuji Film, Tokyo, Japan) to measure glutamic oxaloacetic transaminase (GOT; FUJI DRI-CHEM SLIDE GOT/AST-P III, Fuji Film, Tokyo, Japan), blood urea nitrogen (BUN; FUJI DRI-CHEM SLIDE BUN-PIII, Fuji Film, Tokyo, Japan), glucose (FUJI DRI-CHEM SLIDE GLUPIII, Fuji Film, Tokyo, Japan), albumin (FUJI DRI-CHEM SLIDE ALB-P, FUJIFILM Corp., Tokyo, Japan), and total protein (TP; FUJI DRI-CHEM SLIDE TP-PIII, Fuji Film, Tokyo, Japan).

To determine normal ranges for the CBC data of calves, reference values provided by Abaxis (the IDEXX ProCyte Dx* hematology analyzer, Bovine) and analysis results from Kim et al. (2021) [17] for Hanwoo cattle were used (Table 2 and Table 3).

### 2.5. Statistical Analysis

The statistical analysis of the effects of milk replacer feeding amount and concentration in Hanwoo calves was conducted using the PROC MIXED procedure of SAS (2013, release 9.5 version, SAS Inc., Cary, NC, USA). The model used was

Y_jikl_ = μ + MR_i_ + MC_j_ + IRC_k_ + Al + T_m_ + E_jiklm_

where μ is the overall mean, MR_i_ is the effect of milk replacer feeding amount, MC_j_ is the effect of milk replacer concentration, IRC_k_ is the interaction between milk replacer feeding amount and concentration, A_l_ is the effect of gender, T_m_ is the effect of time, and E_jiklm_ is the experimental error. The fixed effects in the model were milk replacer feeding amount and concentration, while calf gender was considered as a random effect. To clearly distinguish treatment effects and remove time effects, repeated measurement analysis was performed using the CONTRAST option [18]. Multiple comparisons for diarrhea incidence rates based on total milk replacer intake were conducted using the PDIFF option. To evaluate linear and non-linear relationships with total milk replacer intake, orthogonal polynomial contrast, and quadratic line tests were performed using the CONTRAST and NLIN options. Treatment effects were considered significant at *p* < 0.05, and trends were noted at 0.05 ≤ *p* < 0.10. All means are presented as least square means.

## 3. Results

### 3.1. Intake

The effects of milk replacer amount fed and concentration on milk replacer intake, starter feed intake, water intake, and nutrient intake of Hanwoo calves are presented in Table 4. Milk replacer intake was significantly higher in treatment groups fed 20 and 30% of body weight compared with those fed 10% (*p* < 0.001). As the feeding concentration increased from 10 to 25%, milk replacer intake significantly increased in all treatment groups (*p* < 0.001). No significant interaction was observed between feeding level and concentration. Milk replacer intake increased over time in all treatment groups (*p* < 0.001). No gender differences were observed in milk replacer intake across all treatments.

Starter feed intake was higher in the 10% milk replacer feeding group compared with the 20 and 30% groups (*p* = 0.001). No significant differences were observed in starter feed intake based on feeding concentration. There was no significant interaction between milk replacer feeding level and concentration for starter feed intake. Starter feed intake increased over time in all treatment groups (*p* < 0.001). No gender differences were observed in starter feed intake across all treatments.

Water consumption showed no significant differences based on milk replacer feeding levels relative to body weight. However, it increased significantly as the milk replacer concentration increased from 10 to 25% (*p* < 0.01). No significant interaction was observed between feeding level and concentration for water consumption. Water consumption increased significantly over time in all treatment groups (*p* < 0.001). No gender differences were observed in water consumption across all treatments.

### 3.2. Growth Performance and Body Conditions

The effects of milk replacer amount fed and concentration on growth performance and body condition of Hanwoo calves are presented in Table 5. The initial body weight of calves showed no significant differences between treatment groups. Body weight was significantly higher in groups fed 20 and 30% of body weight compared with the 10% group (*p* < 0.001). Milk replacer concentration did not significantly affect body weight across all treatments. In the 30% feeding group, there was a trend towards lower body weight at 25% concentration (*p* = 0.098). Body weight increased over time (*p* < 0.001), with no gender differences observed.

Weight gain and average daily gain were significantly higher in the 20 and 30% feeding groups compared with the 10% group (*p* < 0.001). Milk replacer concentration did not significantly affect weight gain or average daily gain, and no interaction was observed between feeding level and concentration. Both parameters increased significantly over time in all treatment groups (*p* < 0.001), with no gender differences.

Feed efficiency was significantly higher in the 20 and 30% feeding groups compared with the 10% group (*p* < 0.001). Milk replacer concentration did not significantly affect feed efficiency. An interaction between feeding level and concentration was observed, with the 20 and 30% groups showing higher feed efficiency than the 10% group. Feed efficiency increased with concentration in the 10% group but decreased in the 30% group as concentration increased (*p* = 0.002). Feed efficiency increased significantly over time in all treatment groups (*p* < 0.001), with no gender differences.

Body temperature showed no significant differences across treatments, but there was a trend towards higher temperatures in the 20 and 30% feeding groups (*p* = 0.053). A slight decreasing trend in body temperature was observed as concentration increased in these groups (*p* = 0.073). No correlation between feeding level and concentration, time effects, or gender effects was observed for body temperature.

Chest girth was significantly larger in the 20 and 30% feeding groups compared with the 10% group (*p* < 0.001). Milk replacer concentration and the interaction between feeding level and concentration did not significantly affect chest girth. Chest girth increased over time in all calves (*p* < 0.001). Gender did not significantly affect chest girth in any treatment group.

### 3.3. Equation

**Figure 2:**
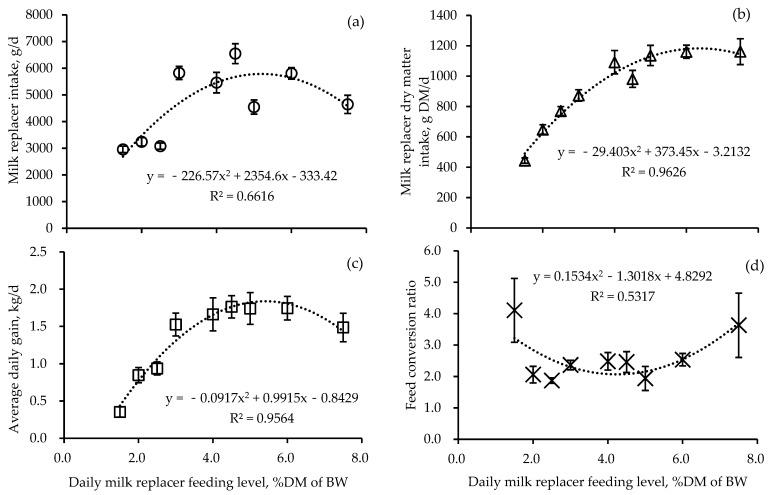
Changes in milk replacer intake (**a**), milk replacer dry matter intake (**b**), average daily gain (**c**), and feed conversion ratio by the daily milk replacer supply quantity in Hanwoo calves (*n* = 10) (**d**). O, milk replacer intake (quadratic effect, *p* < 0.001); Δ, milk replacer dry matter intake (quadratic effect, *p* < 0.001). □, average daily gain (quadratic effect, *p* < 0.001); ×, feed conversion ratio (quadratic effect, *p* < 0.001).

Changes in milk replacer intake, milk replacer dry matter intake, average daily gain, and feed conversion ratio by the daily milk replacer feeding level in Hanwoo calves are presented in Figure 2. As the milk replacer feeding level increased from 1.5% DM of BW to 7.5% DM of BW, milk replacer intake, milk replacer dry matter intake, and average daily gain increased quadratically (Figure 2; *p* < 0.001), and feed conversion ratio decreased quadratically. The equation derived for milk replacer intake based on feeding level relative to body weight is Y = −226.57x^2^ + 2354.6x − 333.42 (Figure 2a, R^2^ = 0.6616, *p* < 0.001). The equation for milk replacer dry matter intake is Y = −29.411x^2^ + 373.58x − 3.4752 (Figure 2b, R^2^ = 0.9626, *p* < 0.001). The equations derived for average daily gain and feed conversion based on feeding level relative to body weight are Y = −0.0917x^2^ + 0.9915x − 0.8429 (Figure 2, R^2^ = 0.9564, *p* < 0.001) and Y = −0.1534x^2^ + 0.6279x − 0.1611 (Figure 2d, R^2^ = 0.5317, *p* < 0.001), respectively.

### 3.4. Diarrhea Incidence

**Figure 3:**
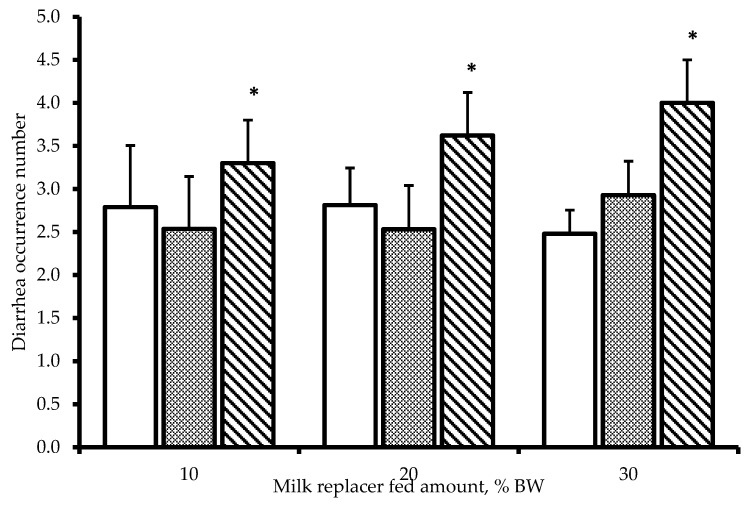
Diarrhea occurrence number according to daily milk replacer fed amount and milk replacer concentration in Hanwoo calves. □, 15% of DM; ▩, 20% of DM; ▧, 25% of DM. * Means significantly different compared to other treatments in the range of 0.05 ≤ *p* < 0.10.3.5. Common Blood Cell.

The diarrhea occurrence number according to daily milk replacer feed amount and milk replacer concentration in Hanwoo calves is presented in Figure 3. No significant differences were observed in diarrhea incidence due to milk replacer feeding levels. However, the incidence of diarrhea occurrences was higher in the 25% concentration group compared with the 15 and 20% concentration groups (*p* < 0.05). There was no interaction observed between milk replacer feeding levels and concentrations.

The CBC results in Hanwoo calves showed significant differences in mean platelet volume (MPV) based on milk replacer feeding level (*p* = 0.003) and concentration (*p* = 0.008). A significant interaction between feeding level and concentration was also observed for MPV (*p* = 0.004). Other blood cell parameters did not exhibit significant differences between treatment groups. However, most blood cell parameters demonstrated significant changes over time (*p* < 0.05) (Table 6).

### 3.5. Blood Metabolites

Effects of milk replacer amount fed and concentration on blood metabolic profiles of Hanwoo calves are presented in Table 7. Among the blood metabolites in Hanwoo calves, glucose levels showed significant differences based on milk replacer feeding levels (*p* = 0.012). However, glutamic oxaloacetic transaminase (GOT), blood urea nitrogen, albumin, and total protein levels did not exhibit significant differences across treatments. Trends were observed in GOT (*p* = 0.093) and albumin (*p* = 0.070) levels in relation to feeding levels and concentrations, respectively.

Temporal effects were evident in blood urea nitrogen (*p* = 0.056), glucose (*p* < 0.001), and albumin (*p* < 0.001) levels. However, GOT and total protein levels did not show significant changes over time.

## 4. Discussion

The National Institute of Animal Science of South Korea recommends feeding 400 g of milk replacer (22.0% CP; 75% TDN) daily to 1–2-week-old calves. Although this guideline does not specify milk replacer concentration, it is equivalent to feeding approximately 13.3% of body weight for a 30 kg calf at 15.0% concentration, which is lower than the minimum treatment group in our study (10 × 15 treatment, 3.0 kg/day). This amount is also considerably less than the average daily milk production of Hanwoo cows, known to be around 2.0–2.5 kg [19]. Milk replacer intake was statistically greatest when fed at 20% of body weight or higher with 20% DM concentration. Generally, as milk replacer or feed intake increases, body weight increases quadratically [20]. Rapid weight gain during the calf stage is known to significantly influence growth during the rearing period, fattening, and post-slaughter meat characteristics. Moreover, nutrients obtained through natural suckling from Hanwoo dams have been reported to be insufficient for actual calf requirements after one month of age. The DM content of Hanwoo cow’s milk is generally known to be around 12.0% [20]. For this reason, most commercial milk replacers recommend feeding at a similar concentration of about 15.0% [21]. In this study, milk replacer intake was lower in the 15% concentration group compared with the 20 and 30% groups. Therefore, if rapid growth is the goal, feeding milk replacers at concentrations of 20% or higher may be strategically advantageous. Numerous studies have shown that feeding large amounts of milk replacer to calves decreases DM feed intake, while feeding less milk replacer increases starter feed intake [8,18]. This study showed similar results, with little correlation to concentration (*p* = 0.881), suggesting that absolute feeding amount was the influencing factor. This implies that excessive milk replacer feeding may hinder the rapid increase in starter feed intake in Hanwoo calves.

A study on dairy calves, where milk replacer dry matter content was fed at five levels ranging from 0 to 25%, reported that as the dry matter content of the milk replacer increased, the dry matter intake by calves increased linearly [22]. The highest average daily gain was observed at a concentration of 15% [21]. In our study, the treatment group that exhibited the highest body weight at 30 days of age and the greatest weight gain over 30 days was the group fed milk replacer at 30% of body weight with a 15% concentration. This result aligns with the findings of the previous research. However, it is well-established that feeding large quantities of feed to cattle at once increases the gastrointestinal passage rate, thereby reducing the contact time between feed and digestive enzymes, which ultimately lowers digestive efficiency [23]. Extrapolated from these findings, feeding too high a concentration of milk replacer to calves may potentially decrease the utilization efficiency of milk replacer in calves.

When feeding livestock, the potential digestibility is influenced by the gastrointestinal passage rate of the feed [24]. In this study, nutritional components were provided in the form of milk replacer, which has a high moisture content. Being liquid, it passes through the digestive system more rapidly compared with solid feeds. A previous study by Pettyjohn et al. [21] demonstrated that as the dry matter content of milk replacer increased, there was a linear decrease in the digestibility of dry matter and crude protein. Extrapolating from these findings, it can be inferred that in the treatment group fed high-concentration milk replacer (25% concentration), the increased gastrointestinal passage rate of the solid components in the milk replacer likely contributed to the observed decreases in weight gain, average daily gain, and feed efficiency. This suggests that while higher concentrations of milk replacer can provide more nutrients per volume, they may also lead to reduced nutrient utilization due to faster transit through the digestive system. The balance between nutrient density and digestibility appears to be a critical factor in optimizing calf growth and feed efficiency.

When animals consume feed, they generate energy through various metabolic processes, during which some energy is released as heat to the environment [25]. Generally, feed intake leads to metabolic heat production. In this study, milk replacer was fed at different levels according to body weight, resulting in varying feed intake across treatment groups. Therefore, the observed differences in body temperature trends among treatment groups can be attributed to the effect of feed intake on metabolic heat production. In this study, calves fed milk replacer at 20 and 30% of body weight showed higher body temperatures compared with those fed at 10% of body weight, which can be attributed to their higher actual feed intake. Interestingly, calves fed milk replacer at 25% concentration tended to have lower body temperatures compared with those fed at 15 and 20% concentrations. Although it did not show clear evidence, this might be correlated with a water intake increase in the 25% treatment group. When calves are exposed to low temperatures and unable to digest consumed feed, it can lead to indigestion, resulting in reduced vitality and decreased body temperature [26]. Although the body temperature decrease observed in this study remained within the normal range and did not indicate disease, this situation is not a positive sign and underscores the need for careful consideration when adjusting feed quantities and concentrations.

Diarrhea is one of the most common problems in calves and, if persistent, can lead to dehydration and additional diseases that may result in death [27]. Calf diarrhea due to reduced small intestinal absorption capacity is known to be caused by villous atrophy, mucosal disorders, intestinal wall amyloidosis, and increased intestinal osmotic pressure from non-absorbable substances [28]. In many cases, the actual cause is the milk replacer or feed being provided.

In this study, as the concentration of milk replacer fed at one time increased, the amount of milk replacer consumed by calves also increased. This suggests that higher milk replacer concentrations may lead to a higher frequency of diarrhea. Treatment groups with higher milk replacer concentrations likely experienced increased diarrhea frequency due to elevated intestinal osmotic pressure caused by larger amounts of milk replacer entering the small intestine [28]. Furthermore, the significant increase in water consumption observed when feeding high-concentration milk replacer to Hanwoo calves (Table 2 and Table 3; *p* < 0.0001) suggests that increased water intake may also contribute to the higher frequency of diarrhea. Therefore, to reduce the frequency of loose stools, including diarrhea, and to provide stable nutrition to calves, it appears ideal to feed milk replacer at concentrations of 20% or lower.

Platelet volume is recognized as an indicator for detecting various conditions, including myocardial infarction, ischemic cerebral infarction, preeclampsia, and arterial stenosis. It is also used as a marker that influences changes in red and white blood cell counts [29]. In clinical research, platelet volume is generally known to increase in the presence of inflammatory bowel disease [29]. However, in our study, all blood cell values remained within the normal range typically observed in Hanwoo cattle [30]. This suggests that the milk replacer feeding levels and concentrations used in this study did not negatively impact blood cell parameters overall. These findings indicate that the milk replacer feeding regimens employed, varying in both quantity and concentration, did not induce significant hematological alterations in Hanwoo calves. This information is valuable for establishing safe and effective feeding protocols that support optimal growth without compromising hematological health in young calves. It is important to note that while subtle variations were observed in some blood parameters, particularly platelet volume, these changes did not exceed normal physiological ranges.

The quantities and concentrations of milk replacer fed in this study were not at levels typically associated with metabolic diseases [31]. Blood glucose is an indicator that increases with a greater intake of non-structural carbohydrates in feed [32]. In our study, the elevated blood glucose levels observed in treatment groups receiving higher amounts of milk replacer likely resulted from increased carbohydrate intake. Glutamic oxaloacetic transaminase (GOT) is an enzyme involved in amino acid formation in animals [33]. It is released in large quantities into the bloodstream when organ cells are damaged, particularly in the liver. Elevated GOT levels can indicate liver damage or fatty liver disease, which is also associated with carbohydrate intake. However, given that the blood metabolite levels and body weights of the Hanwoo calves in this study fell within normal ranges, it appears that the milk replacer quantities and concentrations used across all treatment groups were not sufficient to induce metabolic disorders. Blood urea nitrogen, albumin, and total serum protein are indicators of protein metabolism in livestock [34]. These parameters generally decrease when non-structural carbohydrates, such as starch, are fed in excess [35]. In our study, these three indicators did not appear to be affected by milk replacer quantity or concentration. This suggests that feeding milk replacers at levels approaching the maximum intake capacity of Hanwoo calves does not negatively impact protein metabolism.

In conclusion, the optimal feeding level to maximize calf growth without risking digestive tract disorders due to excessive intake appears to be 4–4.5% of body weight in dry matter. We recommend feeding milk replacer at approximately 20 to 30% of body weight with a concentration between 15 to 20%. This feeding strategy strikes a balance between providing sufficient nutrients for rapid growth and maintaining digestive health. It takes into account the observed effects on weight gain, feed efficiency, and physiological parameters across different treatment groups in our study. By adhering to these guidelines, this result could be used to potentially optimize growth performance while minimizing the risk of nutritional imbalances or digestive issues.

## 5. Conclusions

In Hanwoo calves fed four times daily, feed intake was the greatest for milk replacer levels above 4.0% DM of BW (20% of body weight × 20% milk replacer concentration). The average daily gain was maximized in the treatment group receiving 4.5% DM of BW (30% of body weight × 15% concentration). The 25% milk replacer concentration group showed negative effects on weight gain, average daily gain, and feed efficiency compared with the 15 and 20% groups and also exhibited a relatively higher incidence of diarrhea occurrence. Blood cell parameters and metabolite levels did not show negative effects related to milk replacer feeding levels or concentrations. It is recommended to feed 3.0% DM of BW (20% of body weight × 15% concentration) for the first 10 days, followed by an increase to 4.5% DM of BW (30% of body weight × 15% concentration) after 10 days of age. This feeding strategy aims to optimize growth performance while minimizing potential digestive disturbances in Hanwoo calves during their critical early development stage. Furthermore, this study was conducted to determine the optimal feeding amount and concentration of milk replacer for raising Hanwoo calves and to apply these findings to automatic feeding systems.

## Figures and Tables

**Table 1:** Chemical composition of milk replacer and starter feed.

Item	Milk Replacer ^1^	Starter Feed
DM, %	99.6	88.4
CP, %DM	24.0	25.0
EE, %DM	20.0	3.0
Ash, %DM	8.0	10.0
CF, %DM	NA	10.0
Lactose, %DM	39.4	NA
Ca, %DM	0.7	0.5
p, %DM	0.6	0.5
TDN, %DM	NA	75.0
Lysine, %DM	2.0	NA
Methionine, %DM	0.5	NA
Threonine, %DM	1.2	NA

DM, dry matter; CP, crude protein; EE, ether extract; CF, crude fiber; TDN, total digestible nutrients; NA; not analysis. ^1^ Included Vitamin A, 25,000 IU/kg; Omega 3, 2750 ppm; probiotics, 1 × 10^9^ CFU/kg.

**Table 2:** Treatment arrangement by milk replacer level and concentration of the experiment.

L	10%						20%						30%					
C	15%		20%		25%		15%		20%		25%		15%		20%		25%	
G	F	M	F	M	F	M	F	M	F	M	F	M	F	M	F	M	F	M
N	5	5	5	5	5	5	5	5	5	5	5	5	5	5	5	5	5	5

L, milk replacer level, % of BW; C, milk replacer concentration, %DM; G, gender (F, female; M, male); N, number of animals.

**Table 3:** Reference intervals for the IDEXX ProCyte Dx* hematology analysis (Bovine), pregnant Hanwoo cows and calves ^1^.

Parameter	Unit	Bovine (All Life Stages)	Cows (*n* = 210) ^1^	Calves (*n* = 70) ^1^
RBC	10^6^/µL	4.47–9.35	6.5–10.3	6.8–14.6
HCT	%	22.5–39.9	28.1–46.1	20.4–39.7
Hb	g/dL	7.4–12.8	9.1–15.3	6.5–13.5
MCV	fL	40.4–56.4	34.9–54.3	25.9–35.7
MCHC	g/dL	11.5–18.5	26.5–40.2	25.7–39.2
RDW	%	30.2–33.5	20.6–27.6	21.3–30.0
WBC	10^3^/µL	2.71–17.76	5.2–12.4	4.6–16.4
NE	10^3^/µL	0.68–6.94	1.6–5.8	1.9–10.2
LY	10^3^/µL	1.2–10.62	2.5–6.5	0.9–8.9
MO	10^3^/µL	0.02–2.17	0.06–0.49	0.09–0.84
EO	10^3^/µL	0.01–1.23	0.01–1.16	0.04–1.63
BA	10^3^/µL	0.00–0.04	0.00–0.09	0.00–0.11
PLT	10^3^/µL	147–663	129–573	166–918
MPV	fL	5.9–8.2	5.4–9.3	3.9–6.8

^1^ Kim et al., 2021 [17]. RBC, red blood cell; HCT, hematocrit; Hb, hemoglobin; MCV, mean corpuscular volume; MCHC, mean corpuscular hemoglobin concentration; RDW, red cell distribution width; WBC, white blood cell; NE, neutrophil; LY, lymphocyte; MO, monocyte; EO, eosinophils; BA, basophil; PLT, platelet crit; MPV, mean platelet volume.

**Table 4:** Effects of milk replacer amount fed and concentration on milk replacer intake, starter feed intake, water intake, and nutrient intake of Hanwoo calves for 30 days (*n* = 10).

		Milk Replacer Volume, % of BW × Milk Replacer Concentration, %DM ^1^												
		10			20			30					*p-Value* ^2^	
Items	DAY	15	20	25	15	20	25	15	20	25	SEM	V	C	D
Intakes														
MRI, kg DM	10	0.40	0.59	0.69	0.64	0.84	0.78	0.67	0.77	0.78	0.04	<0.001	<0.001	<0.001
	20	0.45	0.65	0.76	0.90	1.14	1.21	1.07	1.23	1.21	0.05			
	30	0.48	0.71	0.86	1.07	1.30	1.41	1.21	1.48	1.49	0.06			
SI, g DM	10	4.94	9.60	4.45	5.80	2.11	2.35	2.31	2.37	2.00	1.57	0.001	0.881	<0.001
	20	29.6	52.1	47.6	36.7	9.5	13.4	8.8	7.30	7.40	5.55			
	30	56.6	106.2	69.8	48.0	10.7	12.9	12.7	9.50	14.4	8.22			
WI, kg	10	0.15	0.19	0.25	0.14	0.14	0.26	0.10	0.18	0.22	0.04	0.681	<0.001	<0.001
	20	0.19	0.35	0.39	0.26	0.21	0.43	0.18	0.31	0.34	0.07			
	30	0.29	0.45	0.67	0.39	0.44	0.49	0.39	0.40	0.57	0.10			
Nutrient intake														
OMI, kg DM	10	0.36	0.54	0.63	0.59	0.77	0.72	0.61	0.71	0.72	0.028	<0.001	<0.001	<0.001
	20	0.42	0.59	0.70	0.83	1.05	1.12	0.99	1.13	1.11	0.090			
	30	0.44	0.65	0.79	0.99	1.19	1.30	1.11	1.36	1.37	0.103			
CPI, kg DM	10	0.10	0.14	0.17	0.15	0.20	0.19	0.16	0.19	0.19	0.007	<0.001	<0.001	<0.001
	20	0.11	0.15	0.18	0.22	0.27	0.29	0.26	0.30	0.29	0.023			
	30	0.12	0.17	0.21	0.26	0.31	0.34	0.29	0.36	0.36	0.027			
EEI, kg DM	10	0.08	0.12	0.14	0.13	0.17	0.16	0.13	0.15	0.16	0.006	<0.001	<0.001	<0.001
	20	0.09	0.13	0.15	0.18	0.23	0.24	0.21	0.25	0.24	0.020			
	30	0.10	0.14	0.17	0.21	0.26	0.28	0.24	0.30	0.30	0.022			

MR, milk replacer; MRI, milk replacer intake; SI, starter feed intake; WI, water intake; OMI, organic matter intake; CPI, crude protein intake; EEI, ether extract intake; SEM, standard error of the mean. ^1^ Milk replacer volume means the percentage of feeding amount per body weight (1st line), and milk replacer concentration means dry matter content of milk replacer (2nd line). ^2^ V, effect of feed milk replacer volume; C, effect of milk replacer concentration; D, time effect; There was no significant difference in gender effect and the interaction between volume and concentration effect.

**Table 5:** Effects of milk replacer amount fed and concentration on growth performance and body condition of Hanwoo calves for 30 days (*n* = 10).

		Milk Replacer Volume, % of BW × Milk Replacer Concentration, %DM ^1^												
		10			20			30					*p-Value* ^2^	
Items	DAY	15	20	25	15	20	25	15	20	25	SEM	V	C	D
Growth performance														
BW, kg	0	30.6	31.5	30.9	31.1	31.7	30.4	30.9	30.6	30.8	1.19	0.885	0.989	−
	10	29.5	33.2	31.0	34.6	34.2	33.2	34.2	33.8	32.6	1.21	<0.001	0.194	<0.001
	20	31.8	35.9	35.2	40.0	40.4	39.4	40.5	40.9	37.8	1.54			
	30	33.8	40.0	40.3	46.4	48.4	47.4	48.5	48.0	45.6	2.01			
BWG, kg	10	−1.10	1.70	0.11	3.45	2.43	2.86	3.36	3.19	1.81	1.40	<0.001	0.393	<0.001
	20	2.35	2.67	4.20	5.41	6.20	6.16	6.33	7.10	5.16	1.40			
	30	1.93	4.12	5.07	6.39	7.99	8.01	7.95	7.16	7.89	1.40			
ADG, kg/d	10	−0.11	0.17	0.01	0.35	0.24	0.29	0.34	0.32	0.18	0.14	<0.001	0.393	<0.001
	20	0.24	0.27	0.42	0.54	0.62	0.62	0.63	0.71	0.52	0.14			
	30	0.19	0.41	0.51	0.64	0.80	0.80	0.80	0.72	0.79	0.14			
FCR ^3^	10	−7.34	−3.63	−15.88	2.71	3.53	2.68	3.80	2.93	2.18	0.12	<0.001	0.022	0.022
	20	2.53	2.12	1.86	2.04	2.04	−0.30	1.83	2.07	6.48	0.08			
	30	5.68	2.31	1.88	2.36	1.88	1.26	1.75	2.60	2.22	0.08			
Body conditions														
BT, °C	0	38.4	38.6	38.6	38.6	38.9	38.6	38.7	38.7	38.4	0.09	0.053	0.073	0.145
	10	38.6	38.5	38.6	38.8	38.5	38.6	38.8	38.7	38.8	0.09			
	20	38.6	38.6	38.5	38.9	38.7	38.4	38.7	38.6	38.6	0.13			
	30	38.7	38.6	38.6	38.9	38.8	38.6	38.8	38.8	38.7	0.15			
Heart grith, cm	0	72.8	74.6	73.5	74.9	74.7	75.1	73.1	73.2	73.8	0.98	<0.001	0.463	<0.001
	10	74.2	75.3	74.0	75.9	76.7	75.6	75.5	75.2	75.3	1.00			
	20	75.7	77.9	77.6	80.1	80.7	80.0	81.4	80.1	79.3	1.18			
	30	38.4	38.6	38.6	38.6	38.9	38.6	38.7	38.7	38.4	0.09			

BW, body weight; BWG, body weight gain; ADG, average daily gain; FCR, feed conversion ratio; BT, body temperature; SEM, standard error of the mean. ^1^ Milk replacer volume means the percentage of feeding amount per body weight (1st line) and milk replacer concentration means dry matter content of milk replacer (2nd line). ^2^ V, effect of feed milk replacer volume; C, effect of milk replacer concentration; D, time effect; There was no significant difference in gender effect and the interaction between volume and concentration. ^3^ There was a significant difference in the interaction between volume and concentration (*p* = 0.002).

**Table 6:** Effects of milk replacer amount fed and concentration on common blood cell profiles of Hanwoo calves.

	Milk Replacer Volume, % of BW × Milk Replacer Concentration, %DM ^1^													
	10			20			30				*p-Value* ^2^			
Item	15	20	25	15	20	25	15	20	25	SEM	F	C	F × C	D
RBC, M/µL	9.46	9.47	9.35	8.89	9.58	9.25	8.76	8.73	9.0	0.28	0.142	0.680	0.643	0.790
HCT, %	36.9	38.1	36.2	34.1	37.9	35.9	33.1	32.6	36.3	1.25	0.054	0.418	0.133	0.003
Hb, g/dL	11.6	11.7	11.2	10.9	11.7	11.4	10.4	10.4	11.1	0.36	0.070	0.798	0.390	0.043
MCV, fL	37.1	40.3	38.6	46.6	39.5	37.8	36.7	35.4	39.2	2.99	0.350	0.669	0.366	0.390
MCH, pg	11.7	12.4	12.0	12.3	12.2	12.0	11.6	11.2	12.0	0.31	0.068	0.913	0.039	0.031
MCHC. g/dL	30.0	31.0	31.3	32.2	31.0	31.0	30.1	37.2	30.0	2.46	0.656	0.509	0.507	0.907
RDW, %	35.7	36.8	38.3	36.4	36.9	36.2	36.3	36.5	36.0	1.05	0.752	0.823	0.268	<0.001
RETIC, k/ µL	2.21	1.58	2.42	2.19	2.92	2.33	2.15	6.59	3.01	1.25	0.450	0.472	0.604	0.050
WBC, k/µL	9.9	9.8	11.2	36.6	12.2	11.0	10.7	11.0	12.9	8.57	0.409	0.572	0.638	0.073
NE, k/µL	6.73	6.22	7.36	7.25	8.15	7.31	7.13	6.91	8.91	0.72	0.377	0.472	0.459	0.489
LY, k/µL	2.53	2.90	2.89	3.94	3.21	2.73	2.86	3.18	2.99	0.38	0.221	0.428	0.027	<0.001
MO, k/µL	0.47	0.47	0.62	0.67	0.62	0.65	0.55	0.82	0.81	0.12	0.455	0.215	0.643	<0.001
EO, k/µL	0.09	0.11	0.16	0.10	0.08	0.18	0.07	0.13	0.19	0.04	0.147	0.416	0.426	<0.001
BA, k/µL	0.09	0.08	0.11	0.07	0.09	0.08	0.09	0.08	0.12	0.02	0.859	0.028	0.218	<0.001
PLT, k/µL	599.7	579.1	631.9	781.0	649.8	543.4	644.9	599.9	597.5	46.93	0.288	0.741	0.864	<0.001
MPV, fL	8.93	8.98	9.22	9.02	8.96	8.97	8.22	10.46	9.18	0.73	0.003	0.008	0.004	<0.001
PDW, fL	5.96	6.81	5.44	6.74	8.57	4.79	6.56	4.83	7.49	0.97	0.144	0.527	0.141	<0.001
PCT, %	0.57	0.51	0.60	3.31	0.55	0.51	0.57	0.53	0.56	0.89	0.059	0.276	0.426	<0.001

BW, body weight; RBC, red blood cell; HCT, hematocrit; Hb, hemoglobin; MCV, mean corpuscular volume; MCH, mean corpuscular hemoglobin; MCHC, Mean corpuscular hemoglobin concentration; RDW, red cell distribution width; RETIC, reticulocyte; WBC, white blood cell; NE, neutrophil; LY, lymphocyte; MO, monocyte; EO, eosinophils; BA, basophil; PLT, platelet crit; MPV, mean platelet volume; PDW, platelet distribution width; PCT, platelet crit; SEM, standard error of the mean. ^1^ Milk replacer volume means the percentage of feeding amount per body weight (1st line), and milk replacer concentration means dry matter content of milk replacer (2nd line). ^2^ F, effect of fed feed amount; C, effect of milk replacer concentration; F × C, interaction between fed feed amount effect and milk replacer concentration effect; D, effect of time; There was a significant difference shown in the gender effect.

**Table 7:** Effects of milk replacer amount fed and concentration on blood metabolic profiles of Hanwoo calves.

		Milk Replacer Volume × Milk Replacer Concentration ^1^												
		10			20			30					*p-Value* ^2^	
Items	DAY	15	20	25	15	20	25	15	20	25	SEM	V	C	D
GOT, U/L	0	43.6	42.3	46.4	41.0	54.8	51.9	39.6	54.6	48.1	2.17	0.646	0.172	0.285
	10	49.4	41.7	45.7	37.9	50.5	42.5	39.4	44.5	40.3	1.82			
	20	51.1	43.6	46.0	38.6	51.1	41.5	40.7	46.6	37.8	1.37			
	30	46.3	44.6	48.2	39.7	54.6	50.8	45.5	43.8	45.3	1.20			
BUN, mg/dL	0	11.8	8.8	15.1	9.8	11.9	11.1	11.9	12.0	11.5	0.55	0.433	0.098	0.056
	10	16.3	11.0	12.3	8.4	11.3	11.0	9.9	8.9	12.1	0.66			
	20	10.66	7.90	9.30	9.37	6.79	10.15	7.27	7.34	7.68	0.29			
	30	8.78	8.30	8.78	8.52	7.11	24.31	8.12	7.00	8.27	1.66			
Glucose, mg/dL	0	71.9	50.8	78.1	59.2	65.0	59.2	55.6	74.5	82.6	3.65	0.012	0.921	<0.001
	10	93.3	92.6	78.1	91.9	85.8	86.5	104.4	84.0	92.5	2.43			
	20	93.0	95.2	92.1	98.5	115.8	113.1	95.8	113.1	94.0	2.38			
	30	88.6	93.4	88.7	97.0	98.4	118.0	113.0	118.0	108.0	2.71			
Albumin, g/dL	0	2.24	2.29	2.24	2.28	2.29	2.28	2.31	2.31	2.20	0.02	0.988	0.070	<0.001
	10	2.42	2.46	2.39	2.32	2.48	2.34	2.36	2.41	2.28	0.02			
	20	2.54	2.45	2.50	2.40	2.63	2.48	2.55	2.54	2.34	0.03			
	30	2.47	2.65	2.62	2.49	2.68	2.69	2.54	2.65	2.76	0.02			
TP, U/L	0	4.44	4.67	4.72	4.66	5.11	4.86	5.05	4.70	4.38	0.07	0.345	0.291	0.152
	10	4.74	4.86	4.60	4.60	5.04	4.88	5.08	4.71	4.60	0.08			
	20	4.68	4.59	4.47	4.64	4.83	4.56	4.79	4.83	4.32	0.06			
	30	4.67	4.96	4.63	4.56	4.96	4.80	4.75	4.91	4.59	0.05			

GOT, glutamic oxaloacetic transaminase; BUN, blood urea nitrogen; TP, total protein; SEM, standard error of the mean. ^1^ Milk replacer volume means the percentage of feeding amount per body weight (1st line), and milk replacer concentration means dry matter content of milk replacer (2nd line). ^2^ V, effect of feed milk replacer volume; C, effect of milk replacer concentration; D, time effect; There was no significant difference in gender effect and the interaction between volume and concentration.

## Data Availability

No new data were created or analyzed in this study. Data sharing is not applicable to this article.
